# Clinical Characteristics and Predictors of Antiviral Treatment Duration in Hospitalized Patients with Ulcerative Colitis-Associated Cytomegalovirus Colitis, Including Biologic Therapy

**DOI:** 10.3390/v18020188

**Published:** 2026-01-30

**Authors:** Özlem Güler, Hasan Yılmaz

**Affiliations:** 1 Department of Infectious Diseases and Clinical Microbiology, Faculty of Medicine, Kocaeli University, 41001 İzmit, Turkey; 2 Department of Gastroenterology, Faculty of Medicine, Kocaeli University, 41001 İzmit, Turkey; hasan.yilmaz@kocaeli.edu.tr

**Keywords:** ulcerative colitis, cytomegalovirus colitis, antiviral agents, immunosuppressive agents, biological products

## Abstract

Cytomegalovirus (CMV) colitis is a significant entity in hospitalized patients with ulcerative colitis, particularly during immunosuppressive therapy. The factors associated with antiviral treatment duration remain incompletely defined. This retrospective cohort study included hospitalized adult patients with ulcerative colitis and immunohistochemically confirmed CMV colitis. Baseline demographic, clinical, endoscopic, and laboratory characteristics were evaluated for the cohort and stratified by antiviral treatment duration of ≤14 days and >14 days. Correlation analyses were performed between tissue CMV polymerase chain reaction (PCR) viral load and laboratory parameters. Receiver operating characteristic analysis identified a tissue CMV PCR cut-off associated with prolonged antiviral therapy. The study included 52 patients (median age, 41.5 years; 65.4% male). Fourteen patients received biologic therapy and were younger and had higher C-reactive protein levels than those who did not receive biologics. Tissue CMV PCR viral load was higher in patients who received antiviral therapy for >14 days. The analysis identified a tissue CMV PCR cut-off value of 162,000 IU/mg, with an area under the curve of 0.69, sensitivity of 70.4%, and specificity of 76.0%. Tissue CMV PCR viral load showed a weak negative correlation with serum albumin levels (Spearman ’s r = −0.34, *p* < 0.05). Tissue CMV PCR viral load is associated with antiviral treatment duration and may help identify patients with ulcerative colitis–associated CMV colitis who require prolonged therapy.

## 1. Introduction

Cytomegalovirus is a virus with double-stranded DNA that is part of the Herpesviridae family. The seroprevalence of the virus varies according to the geographic region. This virus infects only humans and is typically acquired during childhood, after which it remains in a latent state in the host. Reactivation may occur in immunosuppressed patients, and colonic involvement is often observed [1].

In patients with inflammatory bowel disease, the presence of CMV DNA in colonic tissue has been associated with more severe disease and an increased risk of colectomy and mortality [2]. The use of systemic corticosteroids and other immunosuppressive agents, particularly azathioprine, for ulcerative colitis treatment has been associated with the development of CMV colitis [3]. The effects of biologic agents on CMV colitis appear to vary among patients. The impact of anti-tumor necrosis factor alpha (TNF-α) inhibitors, such as infliximab and adalimumab, on the development of CMV infection remains unclear [1]. However, several studies have reported no significant increase in the risk of CMV infection associated with these drugs. In contrast, Janus kinase inhibitors, including upadacitinib, have been suggested to potentially increase the risk of CMV infection or reactivation [4].

Guidelines differ regarding the optimal timing for initiating antiviral therapy in patients with inflammatory bowel disease and documented CMV infection. Nevertheless, the American College of Gastroenterology (ACG) and European Crohn’s and Colitis Organization (ECCO) recommend initiating antiviral therapy in cases of moderate-to-severe colitis, especially in patients with a high CMV burden in the mucosal tissue [2].

A minimum antiviral treatment duration of at least two weeks is generally recommended, although some patients may require therapy for up to six weeks or longer. In clinical practice, improvement in diarrhea and other symptoms is commonly regarded as a key indicator of response to antiviral treatment [2].

This study aimed to evaluate the clinical characteristics of hospitalized patients with ulcerative colitis who received antiviral therapy for CMV colitis. This study also sought to determine the proportion of patients who received biologic therapy. Although histopathology is sufficient for diagnosing CMV colitis and initiating antiviral therapy, there is no clear guidance on how long to continue antiviral treatment or when it can be safely discontinued. In real-world practice, treatment durations vary widely and are largely based on clinical judgment. Therefore, the factors associated with the duration of antiviral treatment, including clinical, endoscopic, and laboratory findings, were investigated. The analysis focused on identifying differences between patients receiving ≤14 days of antiviral therapy and those requiring more. Furthermore, a 90-day clinical evaluation was conducted to assess disease recurrence, colectomy necessity, and mortality.

## 2. Materials and Methods

This retrospective cohort study was conducted from 2018 to 2025 at a 795-bed tertiary care referral hospital. It was approved by the Ethics Committee of the Kocaeli University Faculty of Medicine under project number 2025/609 and approval code GOKAEK-2025/23/36. The study was designed in accordance with the principles of the Declaration of Helsinki. It was prepared in accordance with the Strengthening the Reporting of Observational Studies in Epidemiology (STROBE) statement [5]. Since patient data were obtained retrospectively from the hospital’s electronic medical record system and anonymized prior to analysis, written informed consent was not obtained. Patient confidentiality was strictly maintained.

The study included adult patients with a diagnosis of ulcerative colitis from different socioeconomic backgrounds who were receiving systemic corticosteroids or other immunosuppressive and biologic therapies. CMV colitis was confirmed by immunohistochemical staining of colonic biopsy specimens, and only patients hospitalized for CMV colitis were enrolled. All patients were hospitalized because of moderate or severe active ulcerative colitis requiring close monitoring, intravenous therapy and managed collaboratively by specialists in infectious diseases and gastroenterology.

Patients were excluded if they were younger than 18 years or had severe systemic conditions associated with immunosuppression, including chronic kidney disease requiring dialysis, rheumatological diseases, or hematologic and solid organ malignancies. Solid organ and hematopoietic stem cell transplant recipients were also excluded. During hospitalization, stool testing using a multiplex gastrointestinal PCR panel on the QIAstat-Dx platform (QIAstat-Dx Gastrointestinal Panel 2; QIAGEN, Hilden, Germany) was performed in all patients to assess the presence of additional infectious enteropathogens. The detection of a secondary infectious gastrointestinal pathogen was defined as an exclusion criterion, thereby reliably excluding coexisting infectious etiologies and ensuring appropriate case selection.

For each patient, demographic and clinical characteristics were documented, including age, sex, duration of ulcerative colitis, disease extent and severity, presence of comorbidities, and use of systemic corticosteroids, other immunosuppressive agents, or biologic therapies within four weeks prior to diagnosis [6]. The use of mesalazine at the time of hospitalization was recorded. Since mesalazine is not associated with an increased risk of CMV colitis and is not recommended for severe ulcerative colitis, it was not considered a factor in determining the duration of antiviral treatment [3]. The extent of the disease was classified according to the Montreal classification, which designates proctitis as category E1, left-sided colitis as category E2, and extensive colitis as category E3 [7]. The Mayo endoscopic subscore, which ranges from 0 to 3, was used to evaluate the endoscopic disease activity. A score of 0 signifies normal or inactive disease, while a score of 1 represents mild disease, characterized by erythema, a reduced vascular pattern, and slight friability. A score of 2 indicates moderate disease, characterized by significant erythema, loss of vascular pattern, friability, and erosions. A score of 3 denotes severe disease with spontaneous bleeding and ulceration. The overall endoscopic severity was further graded using the Ulcerative Colitis Endoscopic Index of Severity (UCEIS), which ranges from 0 to 8 and is composed of three descriptors: vascular pattern, bleeding, and erosions or ulcerations. The vascular pattern is scored from 0 (normal vascular pattern) to 2 (complete loss of vascular pattern); bleeding is scored from 0 (no visible blood) to 3 (luminal moderate or severe bleeding); and erosions and ulcers are scored from 0 (normal mucosa) to 3 (deep ulceration). Higher total scores indicate more severe endoscopic disease activity [8].

In our center, quantitative tissue CMV PCR results are routinely reported to clinicians and are used as part of standard care in patients with moderate-to-severe ulcerative colitis undergoing evaluation for CMV colitis, particularly prior to escalation to biologic therapy. Plasma CMV DNA was not included in the analysis because plasma CMV PCR does not reliably reflect tissue involvement in CMV colitis. Although immunohistochemistry was used for diagnosis, CMV-positive cell counts were not formally quantified and were considered semi-quantitative; therefore, tissue CMV PCR was used to assess viral burden. Tissue cytomegalovirus polymerase chain reaction (PCR) testing was performed on colonic biopsy specimens using a quantitative real-time PCR method, and the numerical viral load value for each patient was recorded. Colonic biopsy specimens were incubated overnight with proteinase K and ATL buffer to achieve tissue liquefaction. DNA was then extracted according to the routine laboratory protocol. CMV DNA was quantified by real-time PCR on the NeuMoDx 96 Molecular System using the NeuMoDx CMV Quant Assay, with run validity ensured by the assay’s controls and calibrators (NeuMoDx Molecular, Inc., a Qiagen company, Ann Arbor, MI, USA) [9]. The assay reports the results as quantitative viral loads in international units per milligram (IU/mg).

In addition to the tissue-based CMV PCR analysis, laboratory parameters obtained at the time of diagnosis were retrospectively reviewed. These included C-reactive protein (CRP), haemoglobin, total leukocyte count with differential, including neutrophils, lymphocytes, monocytes, and eosinophils, platelet count, albumin, lactate dehydrogenase, creatinine, alanine aminotransferase, and aspartate aminotransferase. The selected laboratory parameters were chosen to reflect both ulcerative colitis disease activity and biochemical indicators that are potentially associated with CMV reactivation [10,11].

All patients received antiviral therapy, and details regarding antiviral treatment were recorded. Although trainee physicians are involved in patient care, all decisions regarding antiviral treatment are made in a multidisciplinary setting. This setting includes specialists in gastroenterology and infectious diseases. The duration of antiviral therapy was documented and categorized as ≤14 days or >14 days. Concomitant immunosuppressive treatments were not modified during the antiviral therapy. Treatment-related adverse events, including neutropenia and renal toxicity were documented. During follow-up, clinical outcomes were evaluated, including disease recurrence within 90 days, the requirement for colectomy, and all-cause mortality.

Statistical analyses were performed using IBM SPSS Statistics version 29.0 (IBM Corp., Armonk, NY, USA) and MedCalc Statistical Software version 14 (MedCalc Software Ltd., Ostend, Belgium). The normality of data distribution was assessed using the Kolmogorov–Smirnov and Shapiro–Wilk tests. Continuous variables were expressed as mean ± standard deviation for normally distributed data and as median with interquartile range (25th–75th percentiles) for non-normally distributed data, while categorical variables were presented as numbers and percentages.

Comparisons between groups were performed using the independent samples t-test for normally distributed continuous variables and the Mann–Whitney U test for non-normally distributed continuous variables. Categorical variables were compared using the chi-square test. Group comparisons included patients receiving antiviral therapy for ≤14 days versus >14 days.

Correlation analyses were performed between continuous and ordinal variables, including laboratory parameters and endoscopic activity scores, such as the Mayo endoscopic subscore and Ulcerative Colitis Endoscopic Index of Severity. These analyses were conducted using Spearman’s rank correlation coefficient [12].

Among the variables evaluated for their association with antiviral treatment duration, the tissue CMV PCR viral load was the only parameter found to be statistically significant. Therefore, receiver operating characteristic (ROC) curve analysis was performed for CMV PCR to determine the optimal cut-off value, area under the curve (AUC), and sensitivity and specificity using MedCalc software.

All statistical analyses were two-sided, and a *p*-value of less than 0.05 was considered statistically significant.

## 3. Results

The study included 52 hospitalized patients with ulcerative colitis–associated cytomegalovirus colitis. The median age was 41.5 years (interquartile range [IQR], 29.3–61.8), and 65.4% of patients were male. According to the Montreal classification, extensive colitis (E3) was the most common disease extent, observed in 57.7% of patients, followed by left-sided colitis (E2) in 34.6% and proctitis (E1) in 7.7% of patients.

The endoscopic disease activity was predominantly severe, with 61.5% of patients classified as having severe disease based on the Mayo endoscopic subscore, and 36.5% had moderate disease. The mean Ulcerative Colitis Endoscopic Index of Severity (UCEIS) score was 5.2 ± 1.5. All patients received antiviral therapy with either intravenous ganciclovir or oral valganciclovir, and treatment adherence was good. No major treatment-related adverse events, such as neutropenia or renal failure, occurred during antiviral therapy.

The median tissue CMV PCR viral load was 156,768 IU/mg (with an interquartile range of 30,425–1,013,110). The median duration of antiviral therapy was 21 days (interquartile range, 14–21 days), with treatment durations ranging from 7 to 28 days.

Table 1 summarizes the baseline demographic, clinical, and endoscopic characteristics of the study cohort, and Table 2 presents the laboratory characteristics and factors associated with antiviral treatment duration. Both tables were stratified according to antiviral treatment duration of ≤14 days and >14 days.

Comorbidities were identified in only 13 patients. The most frequently observed comorbidity was hypertension, which was present in nine patients. Five patients had diabetes mellitus, and five had coronary artery disease. Three patients had chronic obstructive pulmonary disease, and one had chronic kidney disease without the need for dialysis.

When patients receiving antiviral therapy for ≤14 days were compared with those treated for >14 days, tissue CMV PCR viral load was the only parameter that differed significantly between the two groups (*p* = 0.019).

**Figure 1:**
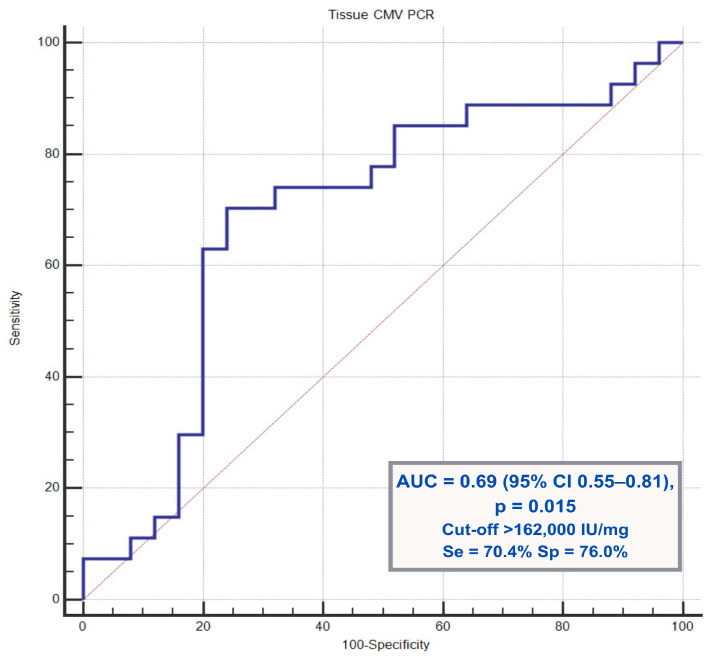
Receiver operating characteristic (ROC) curve of tissue cytomegalovirus (CMV) PCR for predicting prolonged antiviral therapy (>14 days).

Receiver operating characteristic (ROC) curve analysis demonstrated that a tissue CMV PCR viral load cutoff value of >162,000 IU/mg predicted prolonged antiviral treatment with a sensitivity of 70.4%, specificity of 76.0%, and area under the curve (AUC) of 0.69 (*p* = 0.015). Figure 1 shows the ROC curve illustrating the predictive performance of the tissue CMV PCR viral load for antiviral treatment duration.

Only 11 patients were receiving mesalazine at the time of hospitalization. Azathioprine was administered as the non-steroidal immunosuppressive agent. Fourteen patients were receiving biologic therapy. Among patients receiving biologic therapy, infliximab, an anti-TNF agent, was the most frequently used biologic, administered to seven patients (50.0%), followed by adalimumab, another anti-TNF agent, administered to four patients (28.6%) [1]. One patient (7.1%) received ustekinumab, an interleukin-12/23 inhibitor, one patient (7.1%) received vedolizumab, an α4β7 integrin antagonist, and one patient (7.1%) was treated with upadacitinib, a Janus kinase (JAK) inhibitor [13].

Patients receiving biologic therapy were significantly younger than those not receiving it (median age: 30 vs. 43 years; *p* = 0.019). The UCEIS scores and disease durations were similar between the groups. Although the median duration of antiviral therapy was longer in the biologic therapy group, this difference was not significant. Among the laboratory parameters examined, only CRP levels were significantly higher in the biologic therapy group (*p* = 0.030). The tissue CMV PCR viral loads did not differ significantly between the two groups. Table 3 presents the clinical characteristics of patients who received biologic therapy.

**Figure 2:**
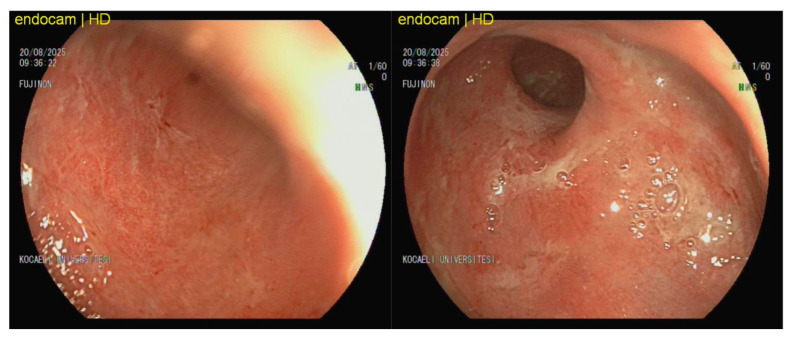
Endoscopic appearance of cytomegalovirus-associated colitis in a 26-year-old male patient with ulcerative colitis receiving biologic and corticosteroid therapy.

Figure 2 depicts the endoscopic findings of a 26-year-old male patient with ulcerative colitis who was receiving infliximab and systemic methylprednisolone at a dosage of 16 mg/day at the time of examination. Despite the administration of biologic and corticosteroid therapies, the distal colon showed signs of diffuse mucosal erythema, granularity, friability, loss of vascular pattern, and superficial mucosal erosions. CMV colitis may present as diffuse erythema, friability, and superficial erosions [14]. The tissue CMV PCR level was markedly elevated at 977,440 IU/mg, consistent with CMV-associated colitis. The patient achieved clinical remission after 21 days of ganciclovir therapy, with no relapse observed at 3 months of follow-up.

**Figure 3:**
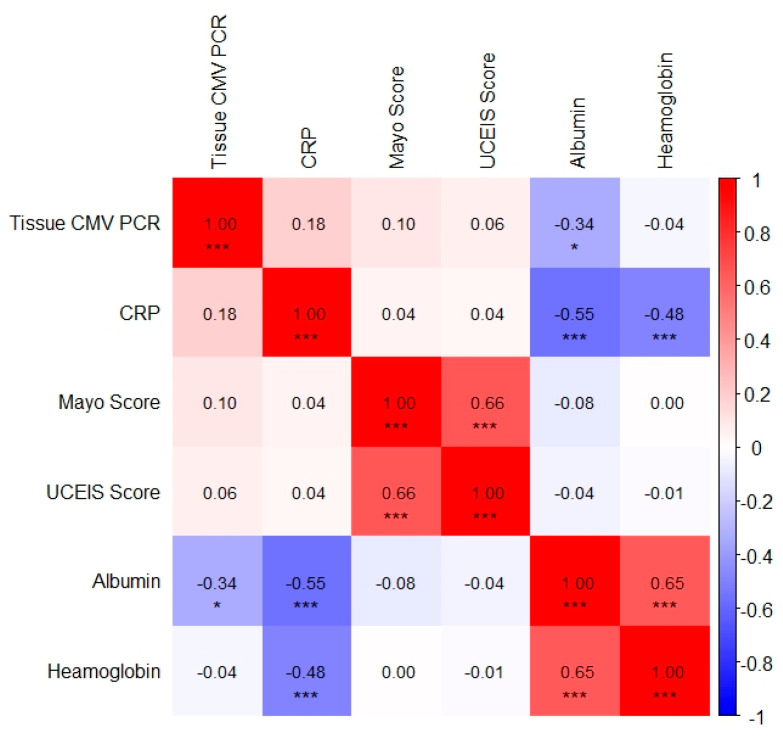
Spearman heatmap graphic showing correlations among clinical, laboratory, and endoscopic parameters. Asterisks denote statistical significance (* *p* < 0.05, *** *p* < 0.001).

Spearman correlation analysis identified the following clinically significant associations. A weak but statistically significant negative correlation was observed between tissue CMV PCR levels and serum albumin levels (r = −0.34, *p* = 0.014). This finding indicates an association between CMV replication and hypoalbuminemia, likely reflecting underlying disease severity and mucosal protein loss. A moderate negative correlation was detected between serum CRP and albumin levels (r = −0.55, *p* < 0.001), which reflected the acute-phase inflammatory response. A moderate positive correlation was found between haemoglobin and albumin levels (r = 0.65, *p* < 0.001), suggesting concurrent inflammatory anemia and protein loss. A moderate negative correlation was also observed between serum haemoglobin and CRP levels (r = −0.48, *p* < 0.001), indicating inflammation-associated anemia. Finally, a moderate positive correlation was identified between the Mayo endoscopic and UCEIS scores (r = 0.66, *p* < 0.001), demonstrating strong concordance between the endoscopic activity indices. The correlations among tissue CMV PCR, inflammatory markers, nutritional parameters, and endoscopic activity indices are summarized in Spearman heatmap graphic (Figure 3).

The patients were observed for three months following the episode of CMV colitis. None of the patients developed toxic megacolon or intestinal perforation, nor did any require colectomy. Two patients died within the three-month follow-up period. The cause of death was *Listeria monocytogenes* encephalitis in one patient and bacterial sepsis in the other patient. Furthermore, eight patients experienced CMV colitis relapse, as confirmed by biopsy.

## 4. Discussion

CMV infection is an important clinical problem that may worsen the disease course in patients with ulcerative colitis. CMV colitis is commonly observed in patients undergoing systemic immunosuppressive therapy, including treatment with modern biologic agents. This study aimed to evaluate the clinical characteristics of patients with CMV colitis and identify the parameters associated with the duration of antiviral treatment.

This study included a patient population predominantly consisting of young individuals with ulcerative colitis and a low burden of comorbidities. Only hospitalized patients with severe clinical presentations were enrolled, forming a relatively homogenous cohort.

The identification of patients with inflammatory bowel disease who are most likely to benefit from antiviral therapy remains controversial. However, antiviral treatment is generally recommended for patients with immunohistochemical positivity and a high viral burden in the colonic tissue. Previous studies have indicated that patients with colonic CMV PCR levels exceeding 250 copies/mg are more likely to respond to antiviral treatment [15]. Furthermore, treatment durations shorter than 14 days have been associated with higher complication and surgery rates in patients with CMV colitis [16].

Current guidelines generally recommend 2–3 weeks of antiviral therapy for the treatment of CMV colitis [17], and both American and European societies suggest a minimum treatment duration of two weeks for patients with ulcerative colitis. However, the optimal timing for treatment discontinuation remains unclear [18,19,20]. Patients with a high CMV DNA load in colonic tissue have been shown to achieve clinical improvement with antiviral therapy alone, without escalating ulcerative colitis–directed treatment [21]. Consistent with these findings, our study demonstrated that a tissue CMV viral load exceeding 162,000 IU/mg was the main determinant of antiviral treatment duration, supporting the role of tissue-based viral burden as a practical marker for guiding treatment decisions. Although antiviral duration was determined by clinical judgement, baseline clinical, endoscopic, and laboratory markers of disease severity were comparable between the two groups. Therefore, the association between viral load and treatment duration is unlikely to be explained simply by more severe disease.

Of the patients in our cohort, 29 were receiving systemic corticosteroids, 24 were receiving azathioprine, and 14 were receiving biological therapies. The relatively low use of biologic therapy in our cohort reflects routine practice in our public tertiary care setting. CMV evaluation is commonly performed before escalating to biologic agents and access may be influenced by cost and national health policies. Corticosteroid exposure alone is a strong risk factor for CMV reactivation in ulcerative colitis. In a large meta-analysis, glucocorticoid use was associated with a more than fourfold increased risk of CMV reactivation (OR 4.175, 95% CI 3.076–5.666) [3]. Although anti-TNF agents alone are not generally considered strong risk factors for CMV reactivation, among patients receiving biological agents, six received concomitant corticosteroids, three received concomitant azathioprine, and only five received biological monotherapy. These findings indicate that most patients were exposed to combined immunosuppression rather than isolated biological therapy and support the concept that the cumulative immunosuppressive burden plays an important factor in the development of CMV colitis [2]. The use of azathioprine, corticosteroids, biologic therapy, and their combinations did not differ between patients treated for ≤14 days and those treated for >14 days. This indicates that immunosuppressive exposure alone did not determine antiviral treatment duration.

Patients receiving biological therapy were significantly younger (median 30 years, IQR 24–52.5; *p* = 0.019) and had higher CRP levels (median 22 mg/L, IQR 10–73; *p* = 0.030). As indicated in previous reports, patients receiving biological therapy tend to be younger and exhibit higher levels of inflammatory markers. Pancolitis and left-sided colon involvement are more prevalent in these patients. Our findings align with those of the current literature on this subject [22].

There were no significant differences in routine laboratory parameters between patients who received antiviral therapy for ≤14 days and those who received longer treatment durations. Eosinopenia and hypoalbuminemia have been reported as potential predictors of CMV colitis in patients with ulcerative colitis; however, no significant differences in routine laboratory values were observed between the two treatment groups in our cohort. However, a weak but statistically significant negative correlation was observed between tissue CMV PCR levels and serum albumin levels (r = −0.34, *p* = 0.014). From a clinical perspective, low serum albumin levels may serve as a practical and easily accessible predictor of CMV colitis, prompting timely tissue-based diagnostic evaluation in hospitalized patients with severe ulcerative colitis [10].

This study has several limitations. First, the retrospective design and relatively small sample size may limit the generalizability of our findings. In addition, routine follow-up colonoscopy was not systematically performed in patients who demonstrated clinical improvement. The timing of post-treatment colonoscopic assessments was not standardized, which may have limited our ability to evaluate the dynamics of mucosal healing.

A major strength of this study is the careful selection of a relatively homogeneous patient population derived from long-term follow-up. Patients were predominantly young and had a low burden of comorbidities, which helped minimize potential confounding factors. In addition, the diagnosis of CMV colitis was based on tissue examination, including molecular testing and immunohistochemical evaluation, allowing for the reliable identification of true CMV colitis. These features strengthen the findings of our study.

CMV colitis is an important and distinct clinical condition in hospitalized patients with severe ulcerative colitis receiving systemic immunosuppressive therapy. This study found that the tissue CMV viral load was the main factor associated with the duration of antiviral treatment. These results support the use of tissue-based molecular testing to guide treatment decisions. Low serum albumin levels may serve as a simple and accessible marker that can raise suspicion of CMV colitis and prompt diagnostic evaluation.

## Figures and Tables

**Table 1:** Baseline demographic, clinical, and endoscopic characteristics of the study population, stratified by antiviral treatment duration.

	Total (*n* = 52)	Antiviral Therapy ≤14 Days (*n* = 25)	Antiviral Therapy >14 Days (*n* = 27)	*p* Value
Age, median (IQR)	41.5 (29.3–61.8)	40 (31–61)	43 (26–62)	0.653 *
Sex, n (%)				0.622 ^†^
Female	18 (34.6)	10 (40)	8 (29.6)	
Male	34 (65.4)	15 (60)	19 (70.4)	
Disease duration, years, median (IQR; min–max)	2 (1–5, 1–15)	2 (1–5, 1–15)	2 (1– 5, 1–12)	0.836 *
Mayo endoscopic subscore, n (%)				0.282 ^†^
Mild	1 (1.9)	0 (0)	1 (3.7)	
Moderate	19 (36.5)	12 (48)	7 (25.9)	
Severe	32 (61.5)	13 (52)	19 (70.4)	
Montreal classification, n (%)				0.762 ^†^
Proctitis (E1)	4 (7.7)	1 (4)	3 (11.1)	
Left sided colitis (E2)	18 (34.6)	9 (36)	9 (33.3)	
Extensive colitis (E3)	30 (57.7)	15 (60)	15 (55.6)	
UCEIS score, mean ± SD	5.2 ± 1.5	5.3 ± 1.4	5.1 ± 1.5	0.548 ^‡^
Systemic corticosteroid use, n (%)	29 (55.8)	13 (52)	16 (59.3)	0.805 ^†^
Other immunosuppressive agents, n (%)	24 (46.2)	12 (48)	12 (44.4)	1 ^†^
Biologic therapy, n (%)	14 (26.9)	5 (20)	9 (33.3)	0.441 ^†^
Azathioprine + Corticosteroids n (%)	14 (26.9)	6 (24)	8 (26.9)	0.885 ^†^

IQR: Interquartile range, SD: Standard deviation, UCEIS: Ulcerative Colitis Endoscopic Index of Severity, * Mann–Whitney U Test, ^†^ Chi-square test, ^‡^ Independent *t*-test.

**Table 2:** Laboratory characteristics of the study population, stratified by antiviral treatment duration.

Laboratory Values	Total (n = 52)	Antiviral Therapy ≤14 Days (n = 25)	Antiviral Therapy >14 Days (n = 27)	*p* Value
Total leucocytes (µL), median (IQR)	7820 (5979.3–10,957.5)	7194 (6199–11,800)	7850 (5700–10,890)	0.869 *
Neutrophil (µL), median (IQR)	5195 (3344.8–7712.5)	5300 (3359.5–8835)	5180 (3320–6800)	0.735 *
Lymphocyte (µL), median (IQR)	1575 (1149.3–2283.5)	1400 (1095–2281.5)	1732 (1149–2287)	0.420 *
Monocyte (µL), median (IQR)	647 (481.3–852)	646 (362–741)	647 (496–880)	0.305 *
Eosinophil (µL), median (IQR)	155 (30–252.3)	160 (15–250.5)	138 (60–260)	0.769 *
Haemoglobin (g/dL), mean ± SD	11 ± 1.9	10.9 ± 2.2	11.3 ± 1.6	0.496 ^†^
Platelet (µL), median (IQR)	303,500 (241,000–383,000)	356,000 (272,500–426,000)	297,000 (215,000–370,000)	0.206 *
AST (U/L), median (IQR)	15 (11–20)	16 (11–20)	15 (11–22)	0.700 *
ALT (U/L), median (IQR)	12 (8–20)	11 (7–19)	13 (8–22)	0.474 *
LDH (U/L), median (IQR)	217 (175–278)	235 (177–289)	195 (175–266)	0.369 *
Albumin (g/L), mean ± SD	33.3 ± 6.3	32.9 ± 5.8	33.7 ± 6.9	0.636 ^†^
Creatinine (mg/dL), median (IQR)	0.7 (0.6–0.9)	0.7 (0.6–0.9)	0.7 (0.6–0.9)	0.776 *
CRP (mg/L), median (IQR)	12 (7–25)	11 (6–37)	14 (8–23)	0.296 *
Tissue CMV PCR, (U/mg), median (IQR)	156,768 (30,425–1,013,110)	55,104 (20,584–197,500)	549,400 (60,352–2,000,000)	0.019 *

IQR: Interquartile range, SD: Standard deviation, CRP: C-reactive protein, CMV: Cytomegalovirus, PCR: Polymerase chain reaction, * Mann–Whitney U Test, ^†^ Independent *t*-test.

**Table 3:** Comparison of clinical characteristics according to biologic therapy use.

Variable	Biologic Therapy (Yes) (*n* = 14)	Biologic Therapy (No) (*n* = 38)	*p* Value
Age, median (IQR)	30 (24–52.5)	43 (33.3–64.3)	0.019 *
Sex, n (%)			0.197 ^†^
Female	7 (50)	11 (28.9)	
Male	7 (50)	27 (71.1)	
Duration of antiviral therapy, days, median (IQR)	21 (14–23)	14 (14–21)	0.228 *
Mayo endoscopic subscore, n (%)			1 ^†^
Mild	1 (7.1)	0 (0)	
Moderate	4 (28.6)	15 (39.5)	
Severe	9 (64.3)	23 (60.5)	
Montreal classification, n (%)			NA
Proctitis (E1)	0 (0)	4 (10.5)	
Left sided colitis (E2)	7 (50)	11 (28.9)	
Extensive colitis (E3)	7 (50)	23 (60.5)	
Systemic corticosteroid use, n (%)	6 (42.9)	23 (60.5)	0.410 ^†^
Other immunosuppressive agents, n (%)	3 (21.4)	21 (55.3)	0.063 ^†^
CRP (mg/L), median (IQR)	22 (10–73)	12 (6–22)	0.030 *
Tissue CMV PCR, (U/mg), median (IQR)	219,760 (36,075–1,148,000)	125,000 (26,003–1,197,500)	0.606 *

IQR: Interquartile range, CRP: C-reactive protein, CMV: Cytomegalovirus, PCR: Polymerase chain reaction, * Mann–Whitney U Test, ^†^ Chi-square test, NA: Not applicable.

## Data Availability

The authors will share the data and materials via email upon request.

## Abbreviations

The following abbreviations are used in this manuscript:

CMV	Cytomegalovirus
PCR	Polymerase chain reaction
UCEIS	Ulcerative Colitis Endoscopic Index of Severity
STROBE	Strengthening the Reporting of Observational Studies in Epidemiology
TNF-α	Tumor necrosis factor alpha
ACG	American College of Gastroenterology
ECCO	European Crohn’s and Colitis Organization
IQR	Interquartile range
ROC	Receiver operating characteristic
AUC	Area under the curve
